# Breastfeeding support during the Covid-19 pandemic in England: analysis of a national survey

**DOI:** 10.1186/s12889-024-20618-2

**Published:** 2024-11-26

**Authors:** Maria A. Quigley, Sian Harrison, Ilana Levene, Phyllis Buchanan, Jenny McLeish, Fiona Alderdice

**Affiliations:** 1 Department of Population Health, NIHR Policy Research Unit in Maternal and Neonatal Health and Care, National Perinatal Epidemiology Unit, University of Oxford, NuffieldOxford, UK; 2 Department of Population Health, National Perinatal Epidemiology Unit, University of Oxford, NuffieldOxford, UK; 3 Breastfeeding Supporter, The Breastfeeding Network, Paisley, UK

**Keywords:** Breastfeeding, Infant feeding, Breastfeeding support, Health professional, Peer support, Covid-19, Pandemic, Survey

## Abstract

**Background:**

Breastfeeding support interventions are associated with longer breastfeeding duration. Contemporary nationally representative data on breastfeeding support as reported by women in England is lacking. Using English national maternity survey data, we describe sources and modes of breastfeeding support as reported by women who gave birth in 2020; sources of support are compared with earlier maternity surveys (2014, 2016, 2018). We also explore the characteristics associated with source/mode of support in 2020 (*n* = 4,611).

**Methods:**

Women who breastfed were asked about sources of breastfeeding support (midwife; other health professional; other formal breastfeeding support such as breastfeeding specialist, breastfeeding support group, peer supporter; and partner/friend/relative), how this help was given and whether they would have liked more help from a health professional with breastfeeding. Adjusted risk ratios (aRR) for the association between sociodemographic and pregnancy-related variables and each source/mode of support were estimated using modified Poisson regression.

**Results:**

From 2014 to 2020 support from midwives and other health professionals declined (from 84.0% to 64.7%, and 61.6% to 15.5% respectively) whereas other formal breastfeeding support and informal support from partners/friends/relatives remained constant at 27–31% and 34–38% respectively. The proportion of women who wanted more help with breastfeeding increased from 30% in 2014–2018 to 46% in 2020. In 2020, women most likely to want more help with breastfeeding were nulliparous (aRR = 1.64, 95%CI:1.50–1.79), younger (aRR = 1.21, 95%CI:1.03–1.42) and of Pakistani ethnicity (aRR = 1.30, 95%CI:1.06–1.60). Receiving breastfeeding support over the phone (35%) was more common than via video call (13%) or text message (5%); these percentages varied according to socio-demographic and pregnancy-related factors.

**Conclusions:**

Breastfeeding support has declined in recent years, and did not meet the needs of many women during the pandemic. Planning for a future emergency should include adequate provision of breastfeeding support particularly if staff are redeployed into other roles. The characteristics associated with support can inform service planning and delivery. Future research should use these factors to develop novel ideas for intervention, such as directly targeting partners or other informal networks with educational or psychosocial interventions.

## Introduction

Breastfeeding support interventions are associated with longer breastfeeding duration [1], and breastfeeding support is recommended as part of national and international guidelines and policies. In England, the NICE postnatal care guideline recommends that breastfeeding care is tailored to women’s individual needs and provides face-to-face support [2]. Written, digital or telephone information should also be offered, including who to contact if additional support is needed, information about peer support, and information for partners.

Effective breastfeeding support is a key intervention to enable women to start and continue breastfeeding, and many factors will determine whether women get the support they need. In England, breastfeeding support in hospital will usually be from the midwife (or neonatal team as appropriate), and following discharge, support will be from the midwife or health visitor. Due to understaffing and lack of resources, support through these routine services may not always be offered, taken up, or meet the needs of women who are learning to breastfeed or experiencing difficulties.

Additional breastfeeding support from trained volunteers or health professionals is available from a variety of services including breastfeeding counsellors, peer supporters and lactation consultants. These services may be provided through hospital clinics, community centres, private practices, and voluntary organisations, and through a variety of modes and frequencies including face-to-face and telephone helplines. Use of these services depends on them being available, accessible, promoted and the woman being motivated to utilise them. Service provision is not standardised, and varies between areas and changes over time, with services regularly being adapted in line with available funding and resources rather than need [3].

The first year of the COVID-19 pandemic and the resultant restrictive measures caused additional challenges and variability in the delivery of breastfeeding support. Women had reduced contact with health professionals, [4–6]; many breastfeeding services were cancelled or reduced, and services that had previously been face-to-face were often delivered ‘remotely’ [7–9]. Remote support encompasses a range of technologies including telephone, video call, text (SMS) messages and mobile applications (apps). Some of these technologies were used before the pandemic; for example, the National Breastfeeding helpline was established in 2008.

Remote breastfeeding support increased markedly during the pandemic [7] and is likely to continue to grow, potentially replacing some face-to-face services in the post-pandemic era. A recent systematic review found that remote breastfeeding support and education combined with support in hospital was effective at increasing exclusive breastfeeding at 3 months, but the effect at other time points and for ‘any’ breastfeeding was less clear [10]. There was also a lack of evidence on maternal satisfaction with remote support. There is little contemporary national data in England on breastfeeding support, particularly remote support, and women’s experiences of this support. This data is needed to inform provision of support both for future pandemic preparedness, but also within the resource constraints and rapidly changing technologies of the post-pandemic era. Using data from national maternity surveys in England, we describe the sources and modes of breastfeeding support as reported by women who gave birth during the pandemic, and compare the sources of support to those reported by women who gave birth before the pandemic (objective 1). We also explore the sociodemographic and pregnancy-related characteristics associated with these sources and modes of support during the pandemic (objective 2).

## Methods

### Design and participants

Data were drawn from the 2020 national maternity survey (NMS) [11]. A random sample of 16,050 women was identified by the Office for National Statistics (ONS) using birth registration records. The inclusion criteria were women who had given birth to their baby in England during a two-week period in May 2020, and who were aged 16 years or older and living in England at the time the birth was registered. May 2020 was chosen so as to identify women who gave birth during the first national lockdown in England. Women were invited to take part at six months postpartum and had the option of completing the questionnaire on paper or online. Women self-reported sociodemographic characteristics and experiences of pregnancy, birth, and the postnatal period, including sections about infant feeding.

### Breastfeeding data

Women were asked whether they ever breastfed their baby (including giving expressed breastmilk), even if only once. Those who answered ‘yes’ were asked additional questions about ‘who or what helped or advised you with breastfeeding your baby’ (hereafter referred to as sources of support), how this help was given (hereafter referred to as mode of support) and whether they would have liked more help from a health professional with breastfeeding their baby. The main sources of support analysed were health professional support (specifically from a midwife or other health professional), other formal breastfeeding support (from a breastfeeding specialist, breastfeeding support group, peer supporter, or other formal support); and informal support (specifically from a partner or friend/relative). The support questions did not distinguish between support given in hospital or after discharge.

### Statistical analysis

All analysis was restricted to women who ever breastfed. As women could select multiple response options for source and mode of support, each source and mode was analysed as a dichotomous variable, and these were described using proportions (objective 1). Where possible we compared these proportions with similar data from previous NMS from 2014, 2016 (which was a pilot study) and 2018 [12–14] to assess the impact of the pandemic on support. The earlier NMS had similar designs to the 2020 NMS, the main difference being that women were sampled at 3 months postpartum in 2014 rather than at 6 months in all other NMS. There were also some differences between the questionnaires in terms of the response options for source of support, and only the 2020 NMS asked about mode of support.

We described the association between key sociodemographic and pregnancy-related variables and each source and mode of support (objective 2). These variables were maternal age, age at completion of full-time education, area deprivation (measured by the Index of Multiple Deprivation, IMD), ethnicity, whether born in/outside UK, parity, mode of birth, hospital length of stay, preterm birth and whether the baby had a neonatal admission. They were chosen because they are strongly associated with breastfeeding variables in this and other studies [15–18]. Modified Poisson regression [19] was used to estimate unadjusted and adjusted risk ratios (aRRs) for these explanatory variables and source/mode of support. The same explanatory variables were included in all models irrespective of p-values thus enabling comparison of associations across different sources or modes of support.

The survey response rate was 29%. Some anonymised sociodemographic information was provided for all women (including non-responders) by ONS. On average response was lower in women who were younger, not married, born outside the UK, living in disadvantaged areas and who had given birth previously [11]. We attempted to correct for these differences using survey weights which were derived using maternal age, marital/registration status, country of birth, region of residence, IMD, and parity. The representativeness of the survey weighted data was assessed by comparing respondent characteristics with data from the ONS source population if available, or from a reference population for England. Around 8.8% of respondents had missing data on explanatory variables and a complete case analysis was employed on the remaining 91.2% of women. All analysis was conducted in Stata version 17 (StataCorp. 2021), using survey-weighted commands to allow for non-response.

## Results

Of the 4,611 respondents to the 2020 survey, 4,009 initiated breastfeeding and 3,658 of these had complete data on explanatory variables and were included in the analysis. Table 1 shows selected characteristics of the 3,658 women included in the analysis and of all 4,611 survey respondents. Following the application of survey weights, the respondents were similar to the reference population for England (all women sampled) in terms of maternal age, IMD, country of birth and parity. When compared with routine data on all births in England, the respondents had a similar prevalence of caesarean birth (29.9% versus 31.2%) and preterm birth (7.5% versus 7.8%). Most minority ethnic groups, particularly Black Caribbean women, were slightly under-represented when compared with women in the 2021 census, while women who self-identified as White Other or Black African were slightly over-represented.

**Table 1:** Characteristics of the women from the 2020 survey included in the analysis

	**Women included in analysis** **(weighted %)** ***N* = 3,658**	**All survey respondents** **(weighted %)** ***N* = 4,611**	**Reference population for England**^**1**^ **(%)**
Age at delivery (years)			
< 25	207 (10.0)	15.3	15.0
25–34	2,200 (59.2)	60.2	60.9
35 +	1,252 (30.8)	24.5	24.2
IMD quintile			
5 most advantaged	853 (17.2)	15.4	15.5
4	872 (18.9)	17.9	17.8
3	760 (19.6)	19.3	19.4
2	690 (21.7)	21.9	22.2
1 least advantaged	483 (22.6)	25.6	25.1
Ethnicity			
White British	2,732 (65.2)	66.3	65.5
White Other	400 (15.4)	13.3	11.1
Indian	120 (3.2)	3.1	3.9
Pakistani	89 (3.3)	2.9	3.4
Bangladeshi	36 (1.3)	1.2	1.5
Black Caribbean	19 (0.8)	0.7	1.2
Black African	74 (4.2)	4.5	3.5
Mixed/other	188 (6.5)	6.3	9.9
Born outside UK	773 (32.2)	30.9	30.4
Nulliparous	1,930 (46.8)	44.3	43.6
Caesarean birth	1,131 (30.0)	29.9	31.2
Preterm	239 (6.9)	7.5	7.8

^1^For age, IMD, country of birth and parity, these percentages are estimated from all 15,972 women sampled (gave birth in England in a two-week period May 2020)

For premature birth and caesarean, these estimates are for all women who gave birth in England in 2019–20: https://www.ons.gov.uk/peoplepopulationandcommunity/birthsdeathsandmarriages/livebirths/bulletins/birthcharacteristicsinenglandandwales/2019. Accessed 21st March 2024: https://digital.nhs.uk/data-and-information/publications/statistical/nhs-maternity-statistics/2019-20. Accessed 21st March 2024

For ethnicity, these percentages are for all women living in England aged 15–49 in the 2021 census: https://www.ons.gov.uk/peoplepopulationandcommunity/culturalidentity/ethnicity/articles/ethnicgroupbyageandsexenglandandwales/census2021. Accessed 21st March 2024

### Breastfeeding support across the surveys 2014–2020

Table 2 shows the sources of breastfeeding support in 2020 and the earlier surveys. Data for 2020 are shown for the 4,009 women who breastfed (to aid comparison with previous surveys; objective 1) rather than for the 3,658 women included in the complete case analysis (objective 2). In all surveys, the most common source of breastfeeding support was the midwife although this was declining across the surveys, even before the pandemic (84% in 2014, 80% in 2016, 70% in 2018, 65% in 2020). There was also a decline in the proportion of women who received support from other health professionals, which again was apparent before the pandemic (62% in 2014, 60% in 2016, 31% in 2018, 15% in 2020). In all surveys, nearly a third of women received other formal breastfeeding support, and just over a third received informal support from their partner, friend or relative. Partner and friend/relative were separate response options in the 2020 survey, and more women received support from a friend/relative than their partner (27% versus 13%). The proportion of women reporting that they were not given any support was 8.2% in 2020 compared with 2–5% in 2014–18. Finally, the proportion of women who wanted more help with breastfeeding from a health professional was 46% in 2020 compared with ~ 30% in 2014–2018.

**Table 2:** Source of breastfeeding support across the surveys 2014–2020

	**2014** ***N* = 3,849**	**2016** ***N* = 489**	**2018** ***N* = 3,999**	**2020** ***N* = 4,009**
Source of support^a^				
*Health professional support*				
Midwife	84.0 (82.7, 85.2)	79.6 (75.1, 83.4)	70.0 (68.4, 71.9)	64.7 (62.9, 66.4)
Other health professional	61.6 (59.9, 63.2)	59.7 (54.6, 64.5)	31.3 (29.8, 33.0)	15.5 (14.2, 16.8)
*Other formal breastfeeding support*^b^	30.6 (29.1, 32.1)	31.5 (27.0, 36.5)	27.2 (25.7, 28.8)	31.0 (29.4, 32.6)
*Informal support*				
*Partner* ~				*13.1* *(12.1, 14.4)*
*Friend/relative* ~				*26.8* *(25.3, 28.4)*
Partner/friend/relative ~	35.2 (33.6, 36.8)	34.3 (29.6, 39.2)	38.5 (36.8, 40.2)	33.7 (32.1, 35.4)
*No support*				
Was not given any support	2.3 (1.8, 2.8)	2.2 (1.1, 4.3)	4.8 (4.0, 5.7)	8.2 (7.2, 9.2)
Did not need any support	7.8 (6.9, 8.7)	11.0 (8.3, 14.5)	13.3 (12.1, 14.7)	10.8 (9.7, 12.0)
Wanted more help from a health professional	29.3 (27.8, 30.8)	31.6 (27.0, 36.7)	30.4 (28.8, 32.1)	46.2 (44.3, 48.0)

Numbers shown are percentages (95% CI)

^a^Source of support response options were not identical across NMS

^b^Includes BF support group (all surveys), peer supporter (all surveys), voluntary organisation (2014 and 2016 only) and BF specialist (2020 only); 2018 and 2020 also have a category ‘other’ where women could write additional detail (these text response have been included)

~ Partner and friend/relative were separate response options in 2020 NMS due to the restrictions on social contact

**Fig. 1:**
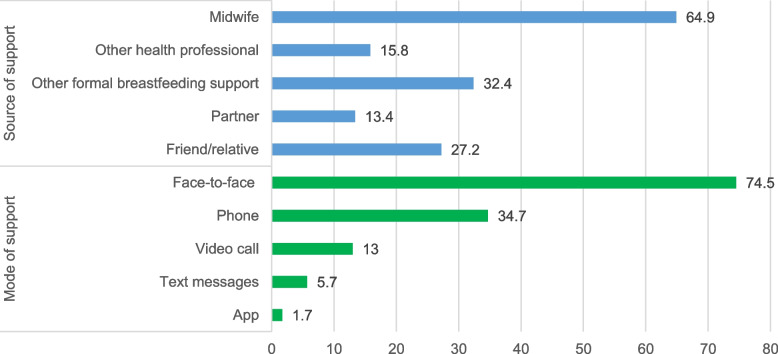
Sources and modes of breastfeeding support in the 2020 NMS

When restricting the 2020 data to women included in the complete case analysis, these figures were almost identical to those in Table 2 (Fig. 1). Among the 3,158 women who received support from a health professional or other formal breastfeeding support in 2020, the majority received some face-to-face support (74.5%, 95% CI: 72.5, 76.3), with support over the phone (34.7%, 95% CI: 32.8, 36.8) and by video call (13.0%, 95% CI: 11.7, 14.4) being the next most common modes of support. Support via text messages (5.7%, 95% CI: 4.8, 6.6) or an app (1.7%, 95% CI: 1.3, 2.3) were uncommon.

### Characteristics associated with breastfeeding support in 2020

Supplementary Figs. 1–10 show the crude prevalence of the breastfeeding support variables by selected sociodemographic and birth–related factors. Figures 2–3 show the aRRs for the associations between these factors and the breastfeeding support variables.

**Fig. 2:**
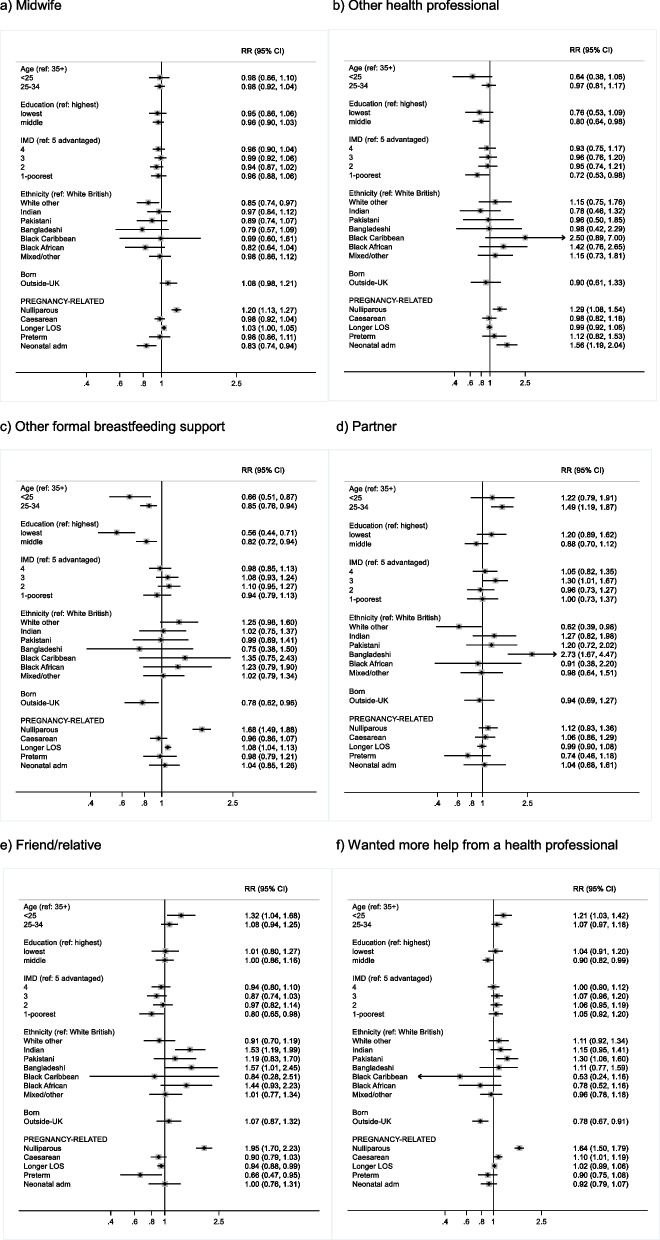
Adjusted risk ratios (RR) for factors associated with reporting support from **a** midwife, **b** other health professional, **c** other formal breastfeeding support, **d** partner, **e** friend/relative, and **f** wanting more help from a health professional in the 2020 NMS. In figure **d**, RR not estimated for Black Caribbean women due to small numbers (0/22)

**Fig. 3:**
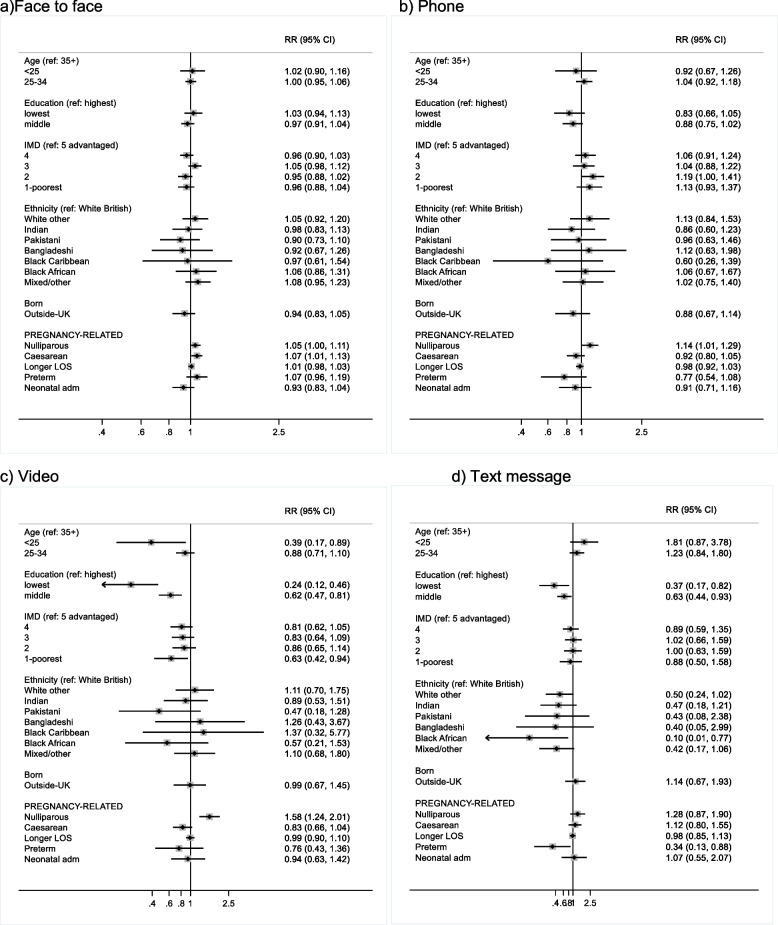
Adjusted risk ratios (RR) for factors associated with reporting different modes of support: **a** face-to-face; **b** phone; **c** video; **d** text message in the 2020 NMS

In multivariable analysis, support from a midwife was more common in nulliparous versus parous women (aRR 1.20) and those with a longer length of stay (aRR 1.03), and was less common in White Other versus White women (aRR 0.85) and when the baby had a neonatal admission (aRR 0.83) (Fig. 2a). Support from another health professional was more common in nulliparous women (aRR 1.29) and when the baby had a neonatal admission (aRR 1.56), and was less common in those who left full-time education aged 17–18 versus 19 years or older (aRR 0.80) (Fig. 2b). Other formal breastfeeding support was more common in nulliparous women (aRR 1.68) and those with a longer length of stay (aRR 1.08), and was less common in younger women (aRR 0.66 for age < 25 and aRR 0.85 for 25–34 versus 35 +) and those who left full-time education aged 16 (aRR 0.56) or 17–18 years (aRR 82) (Fig. 2c).

Informal support from a partner was more common in women aged 25–34 (aRR 1.49), women living in the middle versus most advantaged IMD quintile (aRR 1.30) and Bangladeshi women (aRR 2.73), and was less common in White Other (aRR 0.62) and Black Caribbean women (0/22 reported support from partner) (Fig. 2d). Support from a friend or relative was more common in nulliparous women (aRR 1.95), younger women (aRR 1.32 for age < 25), Indian (aRR 1.53) and Bangladeshi women (aRR 1.57), and was less common in those living in the least advantaged IMD quintile (aRR 0.80), those with a shorter length of stay (aRR 0.94) and a preterm birth (aRR 0.66) and (Fig. 2e).

Wanting more help with breastfeeding from a health professional was more common in nulliparous women (aRR 1.64), those who had a caesarean (aRR 1.10), younger women (aRR 1.21 for age < 25) and Pakistani women (aRR 1.30), and was less common in women born outside the UK (aRR 0.78) and those who left full-time education aged 17–18 (aRR 0.90) (Fig. 2f).

For women who received help from a health professional or other formal breastfeeding support, three quarters (75%) received face-to-face support and this did not vary much across the factors explored: it was slightly more common in nulliparous women (aRR 1.05) and those who had a caesarean versus vaginal birth (aRR 1.07) (Fig. 3a). About a third of women received support over the phone and this did not vary much across the factors explored apart from being more common in nulliparous women (aRR 1.14) (Fig. 3b). Support via a video call varied across the factors explored and was more common in nulliparous women (aRR 1.58) and less common in women aged < 25 (aRR 0.39), women who left full-time education aged 16 (aRR 0.24) or aged 17–18 (aRR 0.62), and those living in the least advantaged IMD quintile (aRR 0.63) (Fig. 3c).

Support via text message was uncommon overall (5%), but was less common in preterm birth (aRR 0.34) and in women who left full-time education aged 16 (aRR 0.37) or aged 17–18 (aRR 0.63), and all ethnic minority groups, particularly White Other (aRR 0.50), Black African (aRR 0.10) and Black Caribbean women (0/22 reported support via text message) (Fig. 3d). Very few women received help via an app (1.8%) and numbers were too small for multivariable analysis.

## Discussion

### Key findings

Our study suggests that breastfeeding support from health professionals has declined in England since 2014 with support from a midwife dropping from four in five women in 2014 to three in five in 2020. The decrease in support from other health professionals was greater, from three in five in 2014 to less than one in five in 2020. Nearly one in three women received other formal breastfeeding support, and one in three received informal support from their partner, friend or relative; these proportions remained constant across 2014–2020. The proportion of women who wanted more help with breastfeeding from a health professional was consistent at around 30% across 2014–2018, but increased markedly to 46% in 2020. For women who received breastfeeding support from a health professional or other formal breastfeeding support in 2020, only 74% received any face-to-face support, 35% received support over the phone and 13% via video, while support via text messages and apps was uncommon.

In the 2020 survey, these figures varied according to sociodemographic and pregnancy-related factors. Nulliparous women were more likely than parous women to receive support from any source – health professional, other formal support, and informal support. Younger mothers were less likely to receive other formal breastfeeding support (for example, breastfeeding specialist, breastfeeding support group, peer supporter), but more likely to receive informal support from friends/relatives. Women who left full-time education earlier and those born outside the UK were also less likely to receive other formal breastfeeding support, whereas women living in the least advantaged areas were less likely to receive support from friends/relatives. Partner support was highest in Bangladeshi women (37%), and support from friends/relatives was highest in Bangladeshi and Indian women (40%).

The groups who were most likely to want more help with breastfeeding from a health professional were nulliparous women, younger mothers and those of Pakistani ethnicity. Compared with UK-born women, those born outside the UK were less likely to want more support. Regarding mode of support, younger mothers, those who left full-time education earlier and those living in the least advantaged areas were less likely to receive support via a video call. Support via text messages was low overall (~ 5%) and lower in all minority ethnic women than in white British women.

### Interpretation of findings

As has been reported in other studies from the UK and elsewhere, before [20, 21] and during the pandemic [22–24], a variety of sources of breastfeeding support were reported by women, including health professional support, other formal support, and informal support from partners and friends/relatives. This likely reflects availability of support which varied geographically especially in the pandemic, and the preferences and needs of individual women. Interestingly, the decline in breastfeeding support from the midwife and other health professionals was not accompanied by an increase in other formal support, or women wanting more help with breastfeeding, until the pandemic (Table 2). Other surveys in England noted a pre-pandemic decline (2017–19) in women having postnatal contact with a midwife as much as they wanted, and a decline (2018–2021) in women receiving help and advice from a midwife or health visitor about feeding in the six weeks after birth [25]. There has been a shortage of midwives in England, together with an increasingly complex caseload, which pre-dated the pandemic [26] and likely affects the amount of time available to support breastfeeding. The decline in support from other health professionals is likely due to the rapid decline in the number of health visitors in England since 2015, which continued during the pandemic [27] and a withdrawal of health visiting services during the pandemic [28]. Support from other health professionals also includes support from the neonatal team, hence the observed association with neonatal admission.

The increase in the proportion of women wanting more support from a health professional during the pandemic may reflect fewer services being available. However, it may also reflect a complex picture of support needs, with some women wanting more support than normal because of other aspects of care or life during the pandemic, or perhaps because more women were more motivated to breastfeed for longer, for example, due to concerns about their baby’s health. Even though there was a decline in breastfeeding support from health professionals, and an increase in women wanting more help with breastfeeding, breastfeeding rates in England [18] and elsewhere in the UK [29–31] were largely unaffected by the pandemic. This may be because there was no decline in other formal breastfeeding support, and this support may have been effective for those who accessed it. Alternatively, the lack of support may have been offset by other pandemic-related factors which had a positive impact on breastfeeding, such as having more uninterrupted time at home to breastfeed. Our data do not reflect the quality of support or the need for support so it is difficult to interpret. For example, nulliparous women were the most likely to want more help with breastfeeding, even though they were more likely to receive help from most sources. This may be partly due to a lack of breastfeeding experience. Women who had a caesarean birth were also more likely to want more help with breastfeeding. As a group, women who have a caesarean birth tend to have more difficulties with breastfeeding and a shorter breastfeeding duration [25, 32]; hence they may benefit from additional support.

Several sociodemographic factors were associated with breastfeeding support. Younger mothers reported wanting more help with breastfeeding than older mothers, and were more likely to receive informal support from a friend/relative than other formal breastfeeding support. Women who left full-time education earlier were also less likely to receive other formal breastfeeding support. Other studies have documented that women who are younger or have lower levels of education may be less likely to access or engage with breastfeeding support organisations [33] such as peer supporters [3, 34], although when they do engage, peer support may be effective in increasing breastfeeding duration [35]. Ensuring that breastfeeding support is targeted at socially disadvantaged groups is particularly important given that these groups tend to have lower rates of breastfeeding in the UK [15–18], and the impact on breastfeeding rates of less support being available may be more marked in these groups [36]. Women born outside the UK were less likely to want more help with breastfeeding than those born in the UK whereas maternal ethnicity showed a more complex pattern. Pakistani women, 43% of whom were born in the UK, were more likely to want more help with breastfeeding than any other ethnic group, both in crude and adjusted analysis. South Asian women, particularly those who were Bangladeshi, were more likely to receive support from their partner or a friend/relative than other ethnic groups. This is consistent with evidence from qualitative studies which have reported that families, particularly grandmothers, are an important source of breastfeeding support among South Asians living in the UK [37–40].

The fact that other formal breastfeeding support varies across sociodemographic factors may partly reflect the needs, motivations, resources and preferences of individual women. However, it will also reflect the availability and equitability of such support across England, which has been shown to be patchy [3], with the availability of face-to-face support services declining even before the pandemic [41]. In contrast, support from a midwife was not strongly associated with any of the sociodemographic factors explored, apart from being slightly lower in women of White Other ethnicity. This suggests that the main barriers to this routine support – such as understaffing and a decline in home visits, which were marked in the pandemic – happened equally across the factors explored. Greater provision of routinely offered breastfeeding support delivered by midwives or health visitors could help reduce inequalities in breastfeeding.

In addition to sources of breastfeeding support, our study looked at mode of support. National policy in England recommends that women receive face-to-face breastfeeding support together with written, digital and telephone support [2]. A quarter of women who received breastfeeding support from a health professional or other formal breastfeeding support did not receive face-to-face support. This is consistent with another survey of women who gave birth in the UK around the same time, in which 21% received no breastfeeding support in hospital [7]. A third of women (35%) in our survey received telephone support whereas support via video call was less common (13%); these percentages are consistent with an online UK survey conducted in 2021 (41% and 12% respectively) [24]. Remote breastfeeding support, particularly over the telephone, was used extensively in the UK before the pandemic, although usually in addition to face-to-face support. The systematic review on remote support combined with postnatal support in hospital found insufficient evidence for higher levels of maternal satisfaction with remote support compared to standard support, although individual studies observed high levels of satisfaction in women who received remote support [10]. High levels of satisfaction have also been observed in women who received breastfeeding support over the phone [42] and via text messages [43].

Remote support has the potential to reduce service costs and improve accessibility, by removing geographical barriers and increasing service availability or flexibility. However, there are potential barriers to the service user and provider, including technology, access costs, language, privacy and personal preference. Some of these barriers have been reported for video calls in the UK and other settings [24], and may explain why, in our study, support via video call was less likely in younger mothers, those who left full-time education earlier and who lived in the poorest areas. Support via text messages in our study was low overall (~ 5%) but lower in all minority ethnic women than in white British women, which may suggest language or cultural barriers. As is the case with face-to-face support, the development and implementation of remote support interventions needs to be multifaceted, or tailored to individual populations so as to maximise access and uptake.

### Strengths and limitations

The main limitation of our study is the low response rate. However, the application of survey weights helped ensure that the respondents were representative of the wider population across key characteristics (Table 1). Most other pandemic surveys are smaller, social media surveys in which white, educated and socially advantaged women are over-represented [7, 22–24], and women were recruited at different points in the postnatal period, up to one year [7, 22] or three years [23], therefore at different stages in their breastfeeding journey. By including women who gave birth in the same month, who were contacted six months postnatally, we have ensured a more homogeneous group of women. We have also interpreted data on sources of support in the pandemic in the context of underlying trends in previous national maternity surveys.

The survey questions on breastfeeding support are relatively crude and provide no measure of frequency or quality of support, and therefore we could not measure overall support across the different sources. We also have no data on what support was available to the women. Some of the sources of support in the survey questionnaire are open to different interpretations, thus giving rise to potential misclassification bias. For example, breastfeeding support groups may be led by health professionals and/or volunteers which make them difficult to classify and for mothers to be certain who provided their support. Our broad classification of ‘other formal breastfeeding support’ may have created a heterogeneous group, hence masking differences. Support from informal sources such as partners is highly subjective and individual women may answer differently depending on the value they place on the emotional, practical, or mechanical aspects of support which can facilitate breastfeeding. While there is good quality evidence on what interventions are effective in a trial setting, and what support is recommended by national policy, there is no population-based data on what support women actually access or receive, and whether they feel it is sufficient. Our study helps to bridge that gap.

## Conclusions

Breastfeeding support has declined in recent years and did not meet the needs of many women during the pandemic. Therefore, we recommend that planning for a future emergency should include adequate provision of breastfeeding support particularly if staff are redeployed into other roles. Some of the characteristics associated with wanting more support, being less likely to receive other formal breastfeeding support, or support via a video call, are markers of socioeconomic disadvantage. Therefore policies aimed at improving routine breastfeeding support, together with local evaluation of breastfeeding support needs and provision, may help meet the support needs in different settings and reduce the stark inequalities in breastfeeding in the post-pandemic era. The factors that we have identified as being associated with support from a variety of sources and delivered in different ways may be used to inform service planning and delivery, including barriers to access for older and newer technologies, and investment in breastfeeding support groups. Future research should use these factors to develop novel ideas for intervention, such as directly targeting partners or other informal networks with educational or psychosocial interventions.

## Data Availability

The data underlying this study is not publicly available because the scope of the consent obtained from study participants restricts our ability to share the data on ethical and legal grounds. There are also third party restrictions by the Office for National Statistics (ONS). Requests to access birth registration data can be submitted to the ONS at https://www.ons.gov.uk/aboutus/whatwedo/statistics/requestingstatistics/makingarequest; information about the ONS data sharing policy can be found at https://cy.ons.gov.uk/aboutus/transparencyandgovernance/datastrategy/datapolicies/onsresearchanddataaccesspolicy. Requests to carry out further analyses on the data from the national maternity surveys can be submitted to the Director of the NPEU at general@npeu.ox.ac.uk. All requests would be subject to the National Perinatal Epidemiology Unit Data Access Policy and may require further regulatory approvals.
